# Stress hyperglycemia ratio and its influence on mortality in elderly patients with severe community-acquired pneumonia: a retrospective study

**DOI:** 10.1007/s40520-024-02831-6

**Published:** 2024-08-22

**Authors:** Lei Miao, Xiaozhu Shen, Zhiqiang Du, Jingxian Liao

**Affiliations:** 1 Department of Geriatrics, The Second People’s Hospital of Lianyungang, Affiliated Hospital of Kangda College of Nanjing Medical University, Lianyungang, Jiangsu China; 2 Department of Critical Care Medicine, The Second People’s Hospital of Lianyungang, Affiliated Hospital of Kangda College of Nanjing Medical University, Lianyungang, Jiangsu China

**Keywords:** Elderly, Severe community-acquired pneumonia, Stress hyperglycemia, Prognosis

## Abstract

**Background:**

Community-acquired pneumonia (CAP) is a significant health issue among the elderly, with severe cases (SCAP) having high mortality rates. This study assesses the predictive significance of the stress hyperglycemia ratio (SHR) in elderly SCAP patients and its impact on outcomes in both diabetic and non-diabetic patients.

**Methods and materials:**

This retrospective study included 406 SCAP patients aged 65 or older from the Second People’s Hospital of Lianyungang (January 2020 to December 2023). Data collected included demographics, medical history, vital signs, and lab results. SHR was calculated from initial blood glucose and estimated average glucose (HbA1c). Statistical analyses, including Cox regression and Kaplan-Meier analysis, evaluated SHR’s impact on mortality. Mediation models explored the effects of neutrophil-lymphocyte ratio (NLR) and SHR.

**Results:**

The 28-day mortality rate was 21.67%. Deceased patients had higher age, Charlson Comorbidity Index, procalcitonin, NLR, glucose, and SHR levels compared to survivors (*P* < 0.05). Both SHR and NLR significantly increased mortality risk, particularly in non-diabetic patients. Combining NLR and SHR improved ROC AUC to 0.898, with 89.80% sensitivity and 81.10% specificity. Kaplan-Meier analysis showed higher cumulative survival for SHR < 1.14, regardless of diabetes status (*P* < 0.05). NLR mediated 13.02% of the SHR-survival relationship, while SHR mediated 14.06% of the NLR-survival relationship.

**Conclusion:**

Elevated SHR is a significant mortality risk factor in elderly SCAP patients, independent of diabetes status. Stringent glucose control and careful monitoring of SHR may improve outcomes in elderly patients with acute respiratory conditions.

**Supplementary Information:**

The online version contains supplementary material available at 10.1007/s40520-024-02831-6.

## Introduction

Community-acquired pneumonia (CAP) is a significant health issue among the elderly, with severe cases (SCAP) having mortality rates as high as 25-50% [1]. Elderly individuals often have underlying conditions that weaken their immune systems, increasing susceptibility to CAP and exacerbating disease severity [2]. Therefore, assessing SCAP severity in clinical practice is crucial. Weakened immune systems and reduced physiological reserves make elderly patients especially prone to SCAP complications. Additionally, these patients often exhibit glucose metabolism disorders, leading to increased complications and mortality.

Recent epidemiological data show that SCAP is an increasing concern among the global elderly population. Studies indicate that SCAP incidence in individuals aged 65 and older is 10 to 30 cases per 1,000 people annually [3]. Moreover, the hospitalization rate for SCAP in this population is rising, significantly impacting healthcare utilization. Despite medical advances, the prognosis for elderly SCAP patients remains poor, highlighting the need for improved diagnostic and treatment strategies.

Stress hyperglycemia is commonly observed in patients with acute illnesses, often due to infections and neuroendocrine disorders [4–6]. Numerous studies have explored the association between the stress hyperglycemia ratio (SHR) and adverse outcomes, such as mortality, length of hospital stay, and complications, emphasizing SHR’s potential utility as a simple and easily obtainable biomarker [7–9]. Patients with severe pneumonia often experience blood glucose fluctuations and develop stress hyperglycemia due to limited oxygen supply [4, 5]. To better evaluate this, SHR combines the initial blood glucose level with an estimated average derived from glycated hemoglobin (HbA1c) [10]. This novel index accounts for baseline glucose levels by utilizing HbA1c, reflecting long-term glycemic control unaffected by acute exacerbations [11].

The relevance of diabetes in the context of SHR and its potential impact on SCAP prognosis is worthy of in-depth study. Diabetic patients, due to long-term hyperglycemia, may respond differently to stress, affecting disease progression and prognosis. Diabetes alters the body’s response to infections and exacerbates the condition through mechanisms like increased inflammatory responses and oxidative stress [12–14]. Studying SHR differences and prognosis between diabetic and non-diabetic SCAP patients is crucial for developing personalized treatment strategies.

This study aims to assess SHR’s predictive significance in elderly SCAP patients and explore its impact on adverse outcomes in both diabetic and non-diabetic patients. By filling this research gap, we hope to provide targeted management strategies to potentially improve outcomes in this vulnerable population.

## Methods

### Study design and patients

The retrospective study was conducted at the Department of Geriatrics, the Second People’s Hospital of Lianyungang affiliated with Kangda College of Nanjing Medical University, from January 2020 to December 2023. Patients aged 65 years or older with a minimum hospital stay of 2 days and meeting the SCAP diagnostic criteria by the Infectious Diseases Society of America/American Thoracic Society (IDSA/ATS) were included [15]. Exclusion criteria were: death within 24 h of admission, hospitalization exceeding 60 days, long-term use of immunosuppressive agents or glucocorticoids, history of acquired immunodeficiency or organ transplantation, undergoing chemoradiotherapy for cancer or hematologic cancer treatment, experiencing diabetic emergencies, or having hospital-acquired infections. The study was approved by the Ethics Committee of the Second People’s Hospital of Lianyungang (NO.2022K040), which waived the requirement for informed consent due to the study’s retrospective, non-interventional, and non-intrusive nature.

### Data collection

Researchers trained in using electronic medical record systems collected comprehensive clinical data. This included timestamps of patient admission and discharge, as well as demographic and medical information such as gender, age, contact details, past medical history, underlying diseases, and vital signs upon admission (temperature, heart rate, respiration rate, blood pressure). Within 24 h of admission, the Charlson Comorbidity Index (CCI) was calculated to assess the presence and severity of comorbidities. CCI calculation involved assigning weighted scores to 19 different medical conditions, with scores ranging from 1 to 6, and summing the assigned scores to obtain an overall index [16].

The Oxygenation Index (OI) was determined using the formula: OI = PaO2/FiO2, where PaO₂ is the arterial partial oxygen pressure and FiO₂ is the inhaled oxygen concentration, providing insights into the patients’ respiratory status [17]. Laboratory tests were performed within the first 24 h of admission, measuring hemoglobin (HB), platelet count (PLT), white blood cell count (WBC), aspartate aminotransferase (AST), alanine aminotransferase (ALT), blood urea nitrogen (BUN), serum creatinine (Scr), serum uric acid (SUA), serum albumin (ALB), procalcitonin (PCT), hypersensitive C-reactive protein (hs-CRP), blood glucose (Glu), and glycosylated hemoglobin (HbA1c). The neutrophil-lymphocyte ratio (NLR) was calculated based on neutrophil and lymphocyte counts.

### Definition and outcome

The stress hyperglycemia ratio (SHR) was calculated based on HbA1c, representing the blood glucose level upon admission divided by the estimated mean blood glucose. The formula is: SHR = Admission Glucose Level / Estimated Average Glucose Level from HbA1c. The calculation of the estimated average glucose level from HbA1c is based on the widely accepted formula: Estimated Average Glucose (mg/dL) = 28.7×HbA1c (%) − 46.7 [10]. In this study, the admission glucose level was defined as the initial plasma glucose concentration within 24 h of admission.

Patients were classified into survival and death groups based on a 28-day follow-up. A comparison was made between the two groups regarding clinical characteristics and SHR levels. Additionally, the impact of SHR on mortality was compared in elderly SCAP patients with and without diabetes. Diabetes was defined as prior treatment with insulin or oral hypoglycemic agents or previously undiagnosed diabetes with HbA1c ≥ 6.5% upon admission.

### Statistical analysis

Statistical analyses were conducted using IBM SPSS Statistics version 21 and PROCESS 4.1. For categorical data, the Chi-squared test was used for group comparisons. The Student t-test was applied to continuous data with normal distribution, expressed as mean ± standard deviation. For quantitative data not conforming to a normal distribution, the non-parametric Mann-Whitney U test was used, expressed as median (quartile) [M (Q1, Q3)]. The Cox proportional hazards regression model was used to evaluate the relationship between the SHR, NLR, and adverse outcomes in SCAP patients. Receiver operating characteristic (ROC) curves and the area under the ROC curve (AUC) were calculated to assess the predictive value of SHR, NLR, and their combined detection. Patients were categorized into two groups based on the cut-off value of SHR. The cut-off value for SHR was determined using ROC curve analysis. Specifically, we used ROC curve analysis to assess the predictive value of SHR for adverse outcomes and selected the cut-off value that maximized sensitivity and specificity. Kaplan-Meier survival analysis was conducted to evaluate the impact of SHR on survival outcomes in patients with or without diabetes, and a log-rank test was used to determine if there was a statistically significant difference in survival between these groups. Using the Bootstrap approach, 5000 repeated samples were analyzed to assess the mediating effect of NLR and SHR. The mediating effect was considered statistically significant if the 95% confidence interval did not include zero. All statistical tests were two-sided, with a significance level set at *P* < 0.05.

## Results

### Patient characteristics

**Fig. 1:**
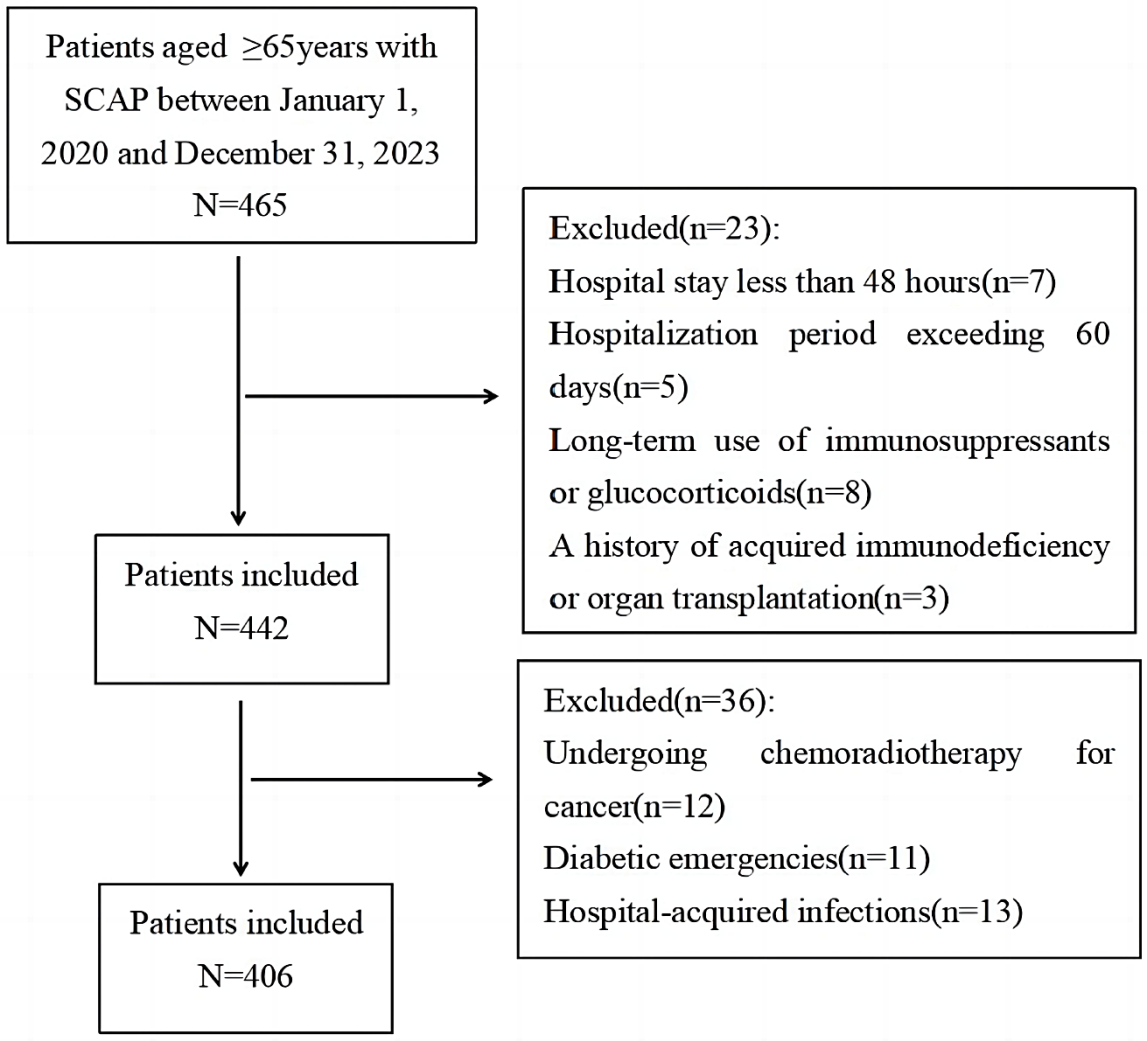
Flow diagram displaying the progress of all participants through the study

The study enrolled a cohort of 406 elderly patients diagnosed with SCAP, adhering to specific inclusion and exclusion criteria as outlined in Fig. 1. The participants had an average age of 80.30 ± 5.26 years, with a 28-day mortality rate of 21.67%. Comparative analysis revealed that the deceased group exhibited significantly higher levels of age, CCI score, PCT, NLR, Glu, and SHR compared to the surviving group ( *P* < 0.05). No significant differences were observed between the two groups regarding gender distribution, prevalence of comorbidities (such as hypertension, diabetes, coronary heart disease, chronic kidney disease, cerebrovascular disease, and chronic lung disease), or etiology ( *P* > 0.05). Additionally, there were no statistically significant differences in various laboratory and clinical parameters, including AST, ALT, Scr, SUA, WBC, PLT, HB, ALB, hs-CRP, HbA1c, HR, SBP, DBP, and OI between the two groups ( *P* > 0.05, Table 1).

**Table 1:** Baseline characteristics of elderly SCAP patients in both groups

Variables	Total (*n* = 406)	Survival group(*n* = 318)	Death group(*n* = 88)	*P*
Male, n (%)	234(57.64)	181(56.92)	53(60.23)	0.627
Age, mean (SD)	80.30(5.26)	80.83(5.15)	83.00(5.31)	0.001
Underlying diseases				
Hypertension, n (%)	195(48.03)	147(46.23)	48(54.55)	0.186
Diabetes, n (%)	152(37.44)	114(35.85)	38(43.18)	0.216
Coronary heart disease, n (%)	136(33.50)	101(31.76)	35(39.77)	0.163
Chronic kidney disease, n (%)	111(27.34)	83(26.10)	28(31.82)	0.283
Cerebrovascular disease, n (%)	132(32.51)	100(31.45)	32(36.36)	0.441
Chronic lung disease, n (%)	135(33.25)	104(32.70)	31(35.23)	0.702
Etiology				
Influenza virus, n (%)	81(19.96)	63(19.81)	18(20.45)	0.881
COVID-19, n (%)	67(16.50)	48(15.09)	19(21.59)	0.148
Bacterial pneumonia, n (%)	147(36.21)	110(34.59)	37(42.05)	0.211
CCI, mean (SD)	2.99(1.20)	2.76(1.10)	3.81(1.17)	< 0.001
AST, U/L, median (IQR)	26.00(22.00,38.00)	26.00(22.00,39.00)	26.00(22.00,35.00)	0.800
ALT, U/L, median (IQR)	23.00(17.00,33.00)	22.00(17.75,33.00)	24.00(17.00,37.00)	0.795
BUN, mmol/L, median (IQR)	7.50(5.98,10.30)	7.30(5.70,10.20)	8.20(6.90,11.85)	0.016
Scr,µmmol/L, median (IQR)	78.50(63.00,117.00)	77.00(63.00,115.00)	86.00(65.50,124.00)	0.106
SUA,µmmol/L, median (IQR)	259.50(202.00,340.00)	257.00(202.00,324.00)	293.00(191.00,363.75)	0.265
WBC, ×109L, mean (SD)	13.01(3.99)	12.88(3.93)	13.48(4.17)	0.212
NLR, median (IQR)	9.93(6.45,13.83)	8.07(6.22,12.38)	19.06(12.74,24.86)	< 0.001
PLT, ×109L, median (IQR)	158.00(125.00,209.00)	157.00(129.00,209.00)	183.00(119.00,210.00)	0.954
HB, g/L, mean (SD)	101.52(22.20)	100.64(22.91)	104.69(19.22)	0.130
PCT, ng/ml, median (IQR)	3.25(1.40,6.49)	2.77(1.21,6.18)	5.10(2.23,10.38)	< 0.001
ALB, g/L, mean (SD)	31.58(3.97)	31.42(3.98)	32.18(3.91)	0.109
hs-CRP, mg/L, median (IQR)	110.00(44.40,162.00)	91.30(44.13,164.70)	121.00(55.40,162.00)	0.491
Glu, mmol/L, median (IQR)	7.90(6.55,10.58)	7.68(6.50,10.47)	8.50(7.70,11.93)	< 0.001
HbA1c,%,median (IQR)	5.86(5.50,7.70)	5.82(5.50,7.70)	6.25(5.43,7.80)	0.620
SHR, median (IQR)	1.12(1.04,1.20)	1.10(1.03,1.15)	1.21(1.15,1.30)	< 0.001
HR, beats/min, median (IQR)	95.00(81.00,111.25)	96.00(78.00,112.00)	95.00(88.25,111.00)	0.432
SBP, mmHg, median (IQR)	129.00(105.00,145.00)	128.00(104.00,145.00)	129.00(109.00,144.00)	0.441
DBP, mmHg, median (IQR)	73.00(62.00,86.00)	73.00(62.00,86.00)	70.50(63.50,84.00)	0.370
OI, mmHg, median (IQR)	316.67(296.67,324.17)	316.67(300.00,323.33)	310.00(296.67,326.67)	0.224

*Abbreviations* COVID-19, coronavirus disease 2019; CCI, Charlson Comorbidity Index, AST, aspartate aminotransferase; ALT, alanine aminotransferase; BUN, Blood urea nitrogen; Scr, Serum creatinine; SUA, Serum uric acid, Hb Hemoglobin; PLT, Platelet count; WBC, White blood cell count; PCT, Procalcitonin; NLR, Neutrophil lymphocyte ratio; hs-CRP, Hypersensitive C-reactive protein; ALB, Serum albumin; Glu, plasma glucose; HbA1c, Glycosylated hemoglobin; SHR, Stress hyperglycemia ratio; HR, Heart rate at admission; SBP, Systolic blood pressure; DBP, Diastolic blood pressure; OI, Oxygenation Index; SD, Standard deviation; IQR, Interquartile range

### Cox proportional hazards regression results

The Cox proportional hazards regression model was used to evaluate the relationship between SHR, NLR, and adverse outcomes in SCAP patients. Three models were employed to understand these relationships across different levels of adjustments for covariates. Model 1: No covariates were adjusted. Model 2: Adjusted for sex, age and CCI. Model 3: Adjusted for age, sex, CCI, underlying diseases, etiology, AST, ALT, BUN, Scr, SUA, WBC, PLT, HB, ALB, hs-CRP, and PCT. Table 2 shows the detailed results and interpretations from each model for both diabetic (DM) and non-diabetic (Non-DM) groups. NLR is significantly associated with increased mortality risk in diabetic patients across all models. For each unit increase in NLR, the risk of mortality increases by approximately 9.1% (Model 1), 9.8% (Model 2), and 15.0% (Model 3), all highly significant with *P* < 0.001. SHR is also significantly associated with increased mortality risk in diabetic patients. For each unit increase in SHR, the risk of mortality increases by approximately 297% (Model 1), 264.6% (Model 2), and 480.3% (Model 3), with consistently significant P-values (*P* < 0.001). NLR is significantly related to increased mortality risk in non-diabetic patients, though the effect is slightly weaker than in diabetic patients. For each unit increase in NLR, the risk of mortality increases by approximately 7.7% (Model 1), 6.3% (Model 2), and 6.1% (Model 3), all statistically significant. SHR is highly predictive of mortality risk in non-diabetic patients. For each unit increase in SHR, the risk of mortality increases dramatically by approximately 1063% (Model 1), 813% (Model 2), and 1006% (Model 3), all with P-values well below 0.001.

**Table 2:** Cox proportional hazards regression results in defferent models

		Model 1 (HR, 95%CI)	*P*	Model 2 (HR, 95%CI)	*P*	Model 3 (HR, 95%CI)	*P*
DM-Group	NLR	1.091 (1.066, 1.117)	< 0.001	1.098 (1.066, 1.131)	< 0.001	1.150 (1.091, 1.211)	< 0.001
	SHR	3.972 (1.725, 9.147)	0.001	3.646 (1.456, 9.133)	0.006	5.803 (1.736, 19.397)	0.004
Non-DM Group	NLR	1.077 (1.043, 1.113)	< 0.001	1.063 (1.025, 1.102)	0.001	1.061 (1.017, 1.107)	0.007
	SHR	11.637 (5.440, 24.891)	< 0.001	9.130 (4.144, 20.114)	< 0.001	11.063 (4.653, 26.300)	< 0.001

Model 1: we did not adjust other covariates

Model 2: we adjust sex, age and CCI

Model 3: we adjust age, sex, CCI, underlying diseases, etiology, AST, ALT, BUN, Scr, SUA, WBC, PLT, HB, ALB, hs-CRP, and PCT

*Abbreviations* CCI, Charlson Comorbidity Index, AST, aspartate aminotransferase; ALT, alanine aminotransferase; BUN, Blood urea nitrogen; Scr, Serum creatinine; SUA, Serum uric acid, Hb Hemoglobin; PLT, Platelet count; WBC, White blood cell count; PCT, Procalcitonin; NLR, Neutrophil lymphocyte ratio; hs-CRP, Hypersensitive C-reactive protein; ALB, Serum albumin; SHR, Stress hyperglycemia ratio; HR, Hazard Ratio; CI, Confidence Interval

### Prognostic value of shr and combined detection

**Fig. 2:**
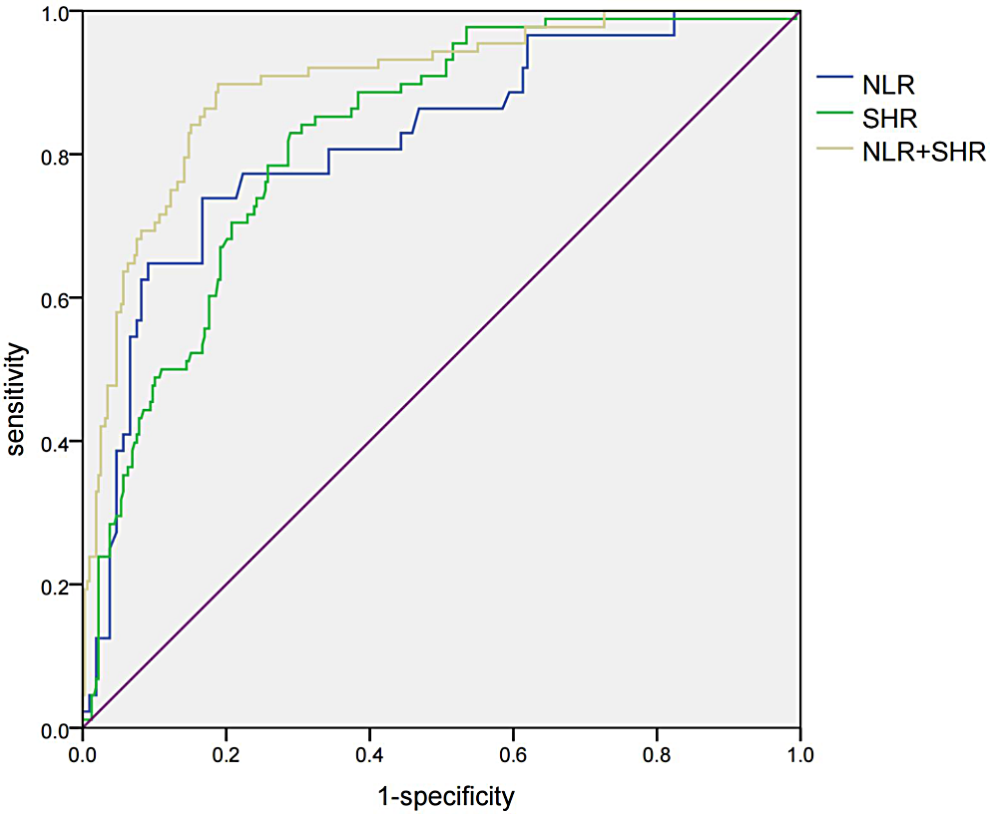
ROC curve of the predictive value ***Abbreviations*** NLR, Neutrophil lymphocyte ratio; SHR, Stress hyperglycemia ratio

Receiver operating characteristic (ROC) curve analysis was conducted to evaluate the predictive value of NLR, SHR, and their combination for adverse outcomes in elderly patients with SCAP. For SHR, the determined cut-off value was 1.14, which achieved the best balance between sensitivity and specificity. The AUC was 0.818 (95% CI: 0.776–0.870) for NLR and 0.823(95% CI: 0.778–0.869) for SHR. For NLR, a cut-off value of 13.28 yielded 73.90% sensitivity and 83.30% specificity. For SHR, a cut-off value of 1.14 resulted in 83.00% sensitivity and 71.10% specificity. Combining NLR and SHR increased the AUC to 0.898 (95% CI: 0.860–0 0.936), providing 89.80% sensitivity and 81.10% specificity ( Fig. 2).

### Kaplan-Meier survival analysis

**Fig. 3:**
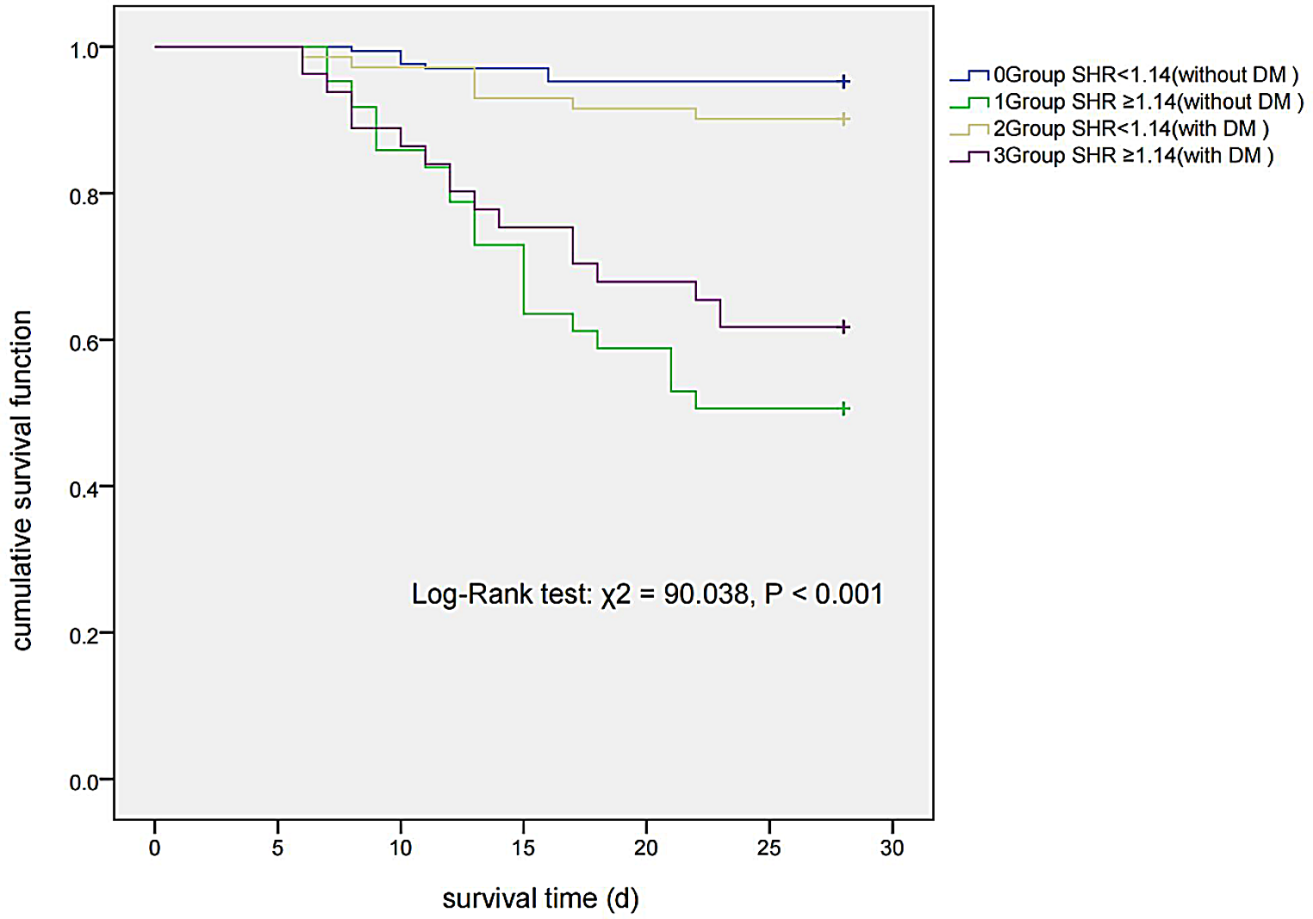
Kaplan-Meier survival curve *Abbreviations* SHR, stress hyperglycemia ratio; DM, diabetes mellitus

Among 254 non-diabetic patients, the group with SHR < 1.14 had 8 deaths out of 169 patients, with a survival time of 27.25 ± 0.26 days and a cumulative survival rate of 95.27%. Conversely, in the group with SHR ≥ 1.14, there were 42 deaths out of 85 patients, with a survival time of 20.88 ± 0.86 days and a cumulative survival rate of 50.59%. For 152 diabetic patients, the group with SHR < 1.14 had 7 deaths out of 71 patients, with an average survival time of 26.54 ± 0.56 days and a cumulative survival rate of 90.14%. In the group with SHR ≥ 1.14, 31 deaths were observed among 81 patients, with an average survival time of 22.38 ± 0.88 days and a cumulative survival rate of 61.73%. A statistically significant difference in cumulative survival was observed between these groups (Log-Rank test: χ² = 90.038, *P* < 0.001) (Fig. 3).

### Simple mediational analyses

A simple mediational model, adjusted for all potential confounders, showed that NLR mediated the relationship between SHR and survival with an indirect effect of 1.831 (95% CI: 0.600–3.471), accounting for 13.02% of the total effect. Similarly, SHR mediated the relationship between NLR and survival with an indirect effect of 0.027 (95%CI: 0.010–0.051), accounting for 14.06% of the total effect (Supplementary Fig. 1). This suggests that NLR and SHR not only independently serve as predictors but also interact with each other.

## Discussion

Community-acquired pneumonia (CAP), particularly its severe form (SCAP), presents a major public health challenge among the elderly due to a combination of factors such as aspiration risk, weakened immune responses, compromised lung function, and pulmonary microangiopathy [2, 18]. Factors that increase susceptibility to infection or exacerbate disease severity significantly impact clinical outcomes. Emerging evidence links stress hyperglycemia, characterized by elevated blood glucose levels, with impaired immune function, and the degree of glycemic disruption reflects the severity of the underlying illness [19, 20]. Stress hyperglycemia can impair lung function, reduce oxygenation capacity, and hinder infection control, leading to prolonged hospital stays and increased mortality [6]. Our study supports this evidence, demonstrating significantly higher initial glucose concentrations in deceased patients compared to survivors.

In recent years, SHR has become a recognized indicator of stress hyperglycemia, accounting for both pre-hospital and post-hospital glucose levels. Studies have established that elevated SHR is associated with adverse outcomes in cardiovascular and cerebrovascular diseases, including higher mortality, longer hospital stays, and increased nosocomial infections [21–23]. Mohammed AQ et al. found that elevated SHR was independently associated with hospitalization for all-cause death, cardiovascular death, and readmission for heart failure [24]. Xu W et al. showed a correlation between SHR and in-hospital mortality in coronary heart disease patients, indicating an SHR index greater than 1.20 increased the risk, especially in pre-diabetes and diabetes mellitus patients [25]. Furthermore, elevated SHR is linked to increased morbidity and mortality following acute myocardial infarction (AMI) [26], and is a predictor for acute kidney injury (AKI), all-cause death, and cardiogenic shock post-AMI [27]. In acute coronary syndrome (ACS) patients, high SHR suggests a higher risk of short-term and long-term adverse outcomes [28]. Our study fills a gap by exploring the relationship between SHR and prognosis in elderly SCAP patients. We reveal significantly higher SHR levels in the deceased group, identifying SHR as a critical mortality risk factor regardless of diabetes mellitus status. This suggests SHR can be a key prognostic indicator for elderly SCAP patients.

This study, through multivariate Cox regression analysis, clearly establishes the clinical significance of the SHR and the NLR as independent prognostic factors in patients with SCAP. Regardless of whether covariates were adjusted, both SHR and NLR significantly increased the risk of mortality. NLR is a significant predictor of mortality in both diabetic and non-diabetic groups. However, the impact appears somewhat stronger in diabetic patients when considering the hazard ratios. SHR exhibits a markedly higher impact on mortality risk, especially in non-diabetic patients. The hazard ratios for SHR are significantly higher in non-diabetic patients across all models, suggesting that SHR plays a crucial role in predicting mortality in this group. Adjusting for additional covariates (from Model 1 to Model 3) generally increases the hazard ratios, which might indicate the presence of confounding factors that attenuate the apparent relationships in unadjusted analyses.These results suggest the need for close monitoring and potential interventional strategies targeting SHR and NLR levels, especially in non-diabetic SCAP patients, to improve clinical outcomes.

Our findings align with Liu B et al., who reported that high SHR in pneumonia patients was associated with increased inflammatory markers, including white blood cells, NLR, C-reactive protein, PCT, and erythrocyte sedimentation rate (ESR) [29]. Consistent with these observations, our study found elevated NLR and PCT levels in deceased patients. Chronic inflammation plays a crucial role in the initiation and progression of diabetes, and chronic hyperglycemia itself can contribute to increased oxidative stress and further stimulate inflammation. Tao J et al. found a strong correlation between elevated SHR levels and an increased risk of stroke-related pneumonia (SAP), particularly among non-diabetic patients [30]. Our study also revealed that SHR and NLR are not only independent prognostic factors but also exhibit interactions with each other. A simple mediation analysis indicated that NLR acts as a mediator in the relationship between SHR and survival, and vice versa. NLR and SHR not only independently serve as prognostic indicators for SCAP patients but also interact with each other. This finding underscores the complex relationship between inflammation and stress responses in critically ill patients. NLR is a crucial indicator of systemic inflammatory response. A high NLR typically indicates a more robust inflammatory reaction, which may influence the overall prognosis of the patients [31]. SHR reflects the hyperglycemic state of the body under stress. Stress hyperglycemia can be driven by multiple factors, including hormone release (such as cortisol and adrenaline) and increased insulin resistance [32]. The interaction between NLR and SHR might reflect the interplay between inflammatory and stress responses in critically ill patients. A high NLR might exacerbate hyperglycemic states and vice versa, potentially worsening the prognosis.

Addressing stress hyperglycemia requires recognizing the complex interaction between comorbidities, inflammation, and glucose dysregulation. Tailored therapeutic strategies to optimize glycemic control may reduce complications and improve outcomes in elderly patients with SCAP. Investing in the development and implementation of management strategies addressing stress hyperglycemia in community-acquired pneumonia could significantly reduce its public health burden, enhance patient outcomes, and improve healthcare efficiency.

In this study, we used the admission blood glucose level to calculate SHR, aligning with the standard methodology used in existing literature. However, it is important to note that blood glucose levels can fluctuate significantly during hospitalization, which may impact patient outcomes. Therefore, examining SHR at multiple time points during hospitalization could offer a more comprehensive and dynamic perspective on hyperglycemia. Specifically, future research could consider calculating SHR at both the initial and final stages of admission to compare their impacts on mortality and other clinical outcomes. This approach could reveal the dynamic changes in stress hyperglycemia throughout the course of the illness and help identify high-risk patient groups, thereby improving clinical intervention strategies. For instance, further studies could explore whether continuous glucose monitoring during hospitalization can more accurately predict patient prognosis and inform personalized treatment plans.

Looking forward, future research directions should focus on the development of personalized treatment protocols aimed at better managing stress hyperglycemia in elderly patients with CAP. Additionally, investigating the molecular mechanisms underlying the relationship between hyperglycemia and immune response could unlock new therapeutic targets. The integration of artificial intelligence and machine learning to predict patient outcomes based on SHR and other biomarkers may also provide a more precise approach to managing and ultimately improving the prognosis of elderly patients with SCAP.

This retrospective study, conducted at a single center with a limited sample size, offers valuable insights into the impact of the stress hyperglycemia ratio (SHR) on the prognosis of patients with community-acquired pneumonia (CAP). However, the study’s design presents several limitations. The single-center nature of the study may limit the generalizability of the findings to broader populations. Additionally, the relatively small sample size restricts the statistical power and the ability to detect more subtle effects. The lack of etiological classification of CAP in our study is another limitation, as the influence of different pathogens on the observed outcomes remains unclear. Potential selection bias also exists, as the study population may not be fully representative of all elderly patients with CAP.

To address these limitations, future large-scale, multicenter prospective studies are necessary to validate and expand upon our findings. Such studies should aim to include a more diverse patient population and provide a detailed etiological classification of CAP. Furthermore, exploring other indicators of blood glucose variation, such as time in target blood glucose range and glucose variability, could provide a more comprehensive understanding of glycemic control’s impact on long-term outcomes and quality of life in critically ill elderly patients.

## Conclusions

This study highlights that an increased SHR is a significant risk factor for mortality in elderly patients with SCAP, irrespective of the presence of diabetes. The pivotal impact of SHR on clinical outcomes underscores its importance as a potentially modifiable risk factor. Managing SHR levels through stringent glucose control and meticulous monitoring may lead to an improved prognosis for elderly patients with acute respiratory conditions.

These findings emphasize the need for a comprehensive approach to patient care, where clinicians must closely monitor and manage SHR levels alongside other clinical parameters to optimize treatment strategies. By integrating SHR management into routine clinical practice, healthcare providers can enhance the overall survival and well-being of the elderly SCAP population. Future research should focus on developing tailored therapeutic protocols and investigating the underlying mechanisms linking hyperglycemia and immune response. Additionally, the potential of advanced predictive analytics, such as artificial intelligence and machine learning, in optimizing glycemic control and improving patient outcomes warrants further exploration.

## Electronic supplementary material

Below is the link to the electronic supplementary material.

Supplementary Material 1: Simple mediational analyses *Abbreviations* NLR, Neutrophil lymphocyte ratio; SHR, Stress hyperglycemia ratio.

## Data Availability

All the data will be available from the corresponding author upon reasonable request.
