# Supplementary figures and images for: Stress hyperglycemia ratio and its influence on mortality in elderly patients with severe community-acquired pneumonia: a retrospective study
Source: Aging Clin Exp Res. 2024 Aug 22;36(1):175. doi: 10.1007/s40520-024-02831-6 (PMC11341645; doi:10.1007/s40520-024-02831-6)

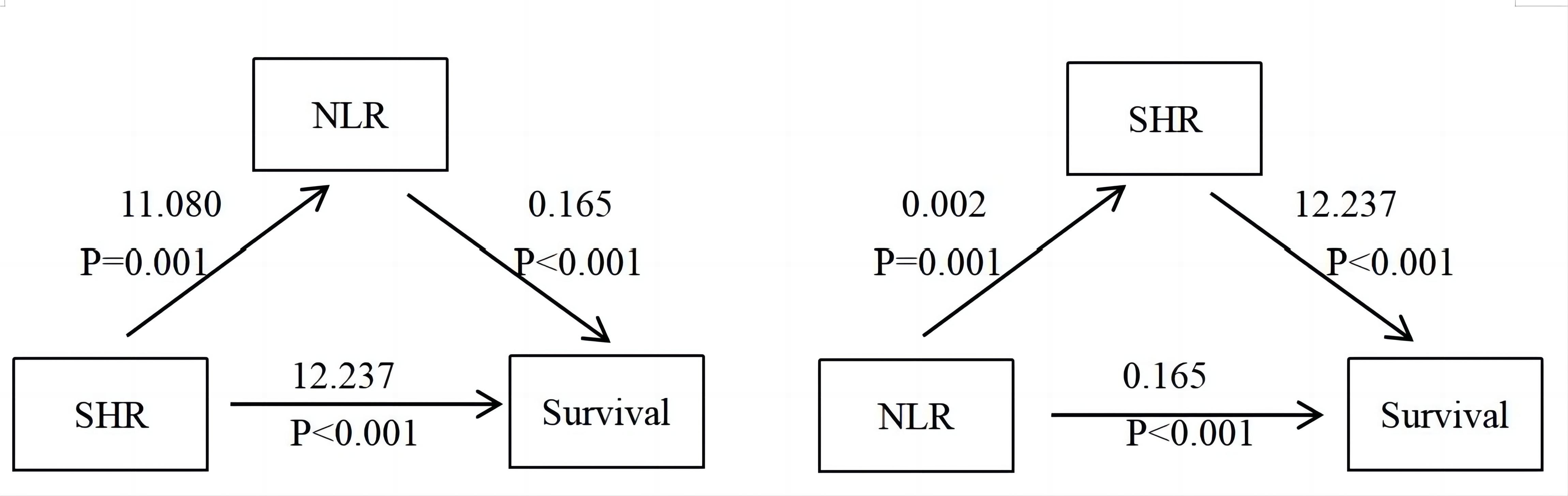

Supplement: Supplementary file 1 — Supplementary Material 1: Simple mediational analyses Abbreviations NLR, Neutrophil lymphocyte ratio; SHR, Stress hyperglycemia ratio. [file 40520_2024_2831_MOESM1_ESM.jpg]
